# Parathyroid adenoma causing spontaneous cervical hematoma: two case reports

**DOI:** 10.1186/s13104-015-1611-0

**Published:** 2015-11-26

**Authors:** Hitomi Shinomiya, Naoki Otsuki, Shin-ichi Takahara, Rie Yasui, Naoki Sawada, Hirokazu Komatsu, Hisami Fujio, Hajime Fujiwara, Ken-ichi Nibu

**Affiliations:** Department of Otolaryngology-Head and Neck Surgery, Kobe University Graduate School of Medicine, 7-5-1 Kusunoki-Cho, Chuo-Ku, Kobe, 6500017 Japan; Department of Otorhinolaryngology, Kakogawa West City Hospital, Kakogawa, Japan; Department of Otolaryngology, Nishi-Kobe Medical Center, Kobe, Japan

**Keywords:** Cervical hemorrhage, Parathyroid adenoma, Cervical swelling, Mediastinal mass, Ecchymosis

## Abstract

**Background:**

Although spontaneous rupture of a cervical parathyroid adenoma with extracapsular hemorrhage is rare, it may cause cervical and mediastinal hematoma, leading to potentially fatal consequences.

**Case presentation:**

The first case was a 76-year-old Asian female who presented with pharyngeal discomfort and anterior chest ecchymosis. Endoscopic investigation showed submucosal hemorrhage in the pharynx and larynx. The second case was a 62-year-old Asian male who presented with anterior chest ecchymosis and suspected of a ruptured blood vessel. Both cases were diagnosed parathyroid adenoma with extracapsular bleeding by hypercalcemia, high levels of intact parathyroid hormone and presence of a nodule behind the thyroid. Both cases were treated with excision of tumor 7 months after initial presentation. After surgery, serum calcium and parathyroid hormone levels had decreased to normal level in both cases.

**Conclusion:**

Extracapsular bleeding of a parathyroid adenoma should be considered in the differential diagnosis of non-traumatic neck hematoma.

## Background

Primary hyperparathyroidism, usually caused by a single functional parathyroid adenoma, was found to occur in 0.2–0.5 % of a Swedish population [1]. Parathyroid adenomas usually come to clinical attention due to overexpression of parathyroid hormone (PTH) [2], which leads to hypercalcemia. The symptoms of excess PTH are subject to extreme variability. Some patients may remain asymptomatic, whereas others may lead to osteoporosis, kidney stones and rarely develop acute and severe dehydration resulting in coma as a manifestation of hypercalcemic parathyroid crisis [3, 4]. Other manifestations may include systemic symptoms of depression and fatigue [3–5].

Although very rarely, parathyroid adenoma can cause extracapsular bleeding. In 1934, Capps first reported a case of massive hemorrhage secondary to rupture of a parathyroid adenoma [6]. Spontaneous extracapsular hemorrhage may lead to narrowing of the airway, necessitating emergency intervention. It can also cause acute painful neck swelling, cervical ecchymosis, hoarseness and dysphagia due to compression of surrounding organs such as the trachea, recurrent nerve and esophagus. If this phenomenon occurs within the mediastinum, the bleeding can mimic aortic dissection.

Here we report two cases of spontaneous extracapsular bleeding from parathyroid adenoma with anterior chest wall ecchymosis. To our knowledge, only 35 such cases have been documented to date.

## Case presentation

### Case 1

**Fig. 1:**
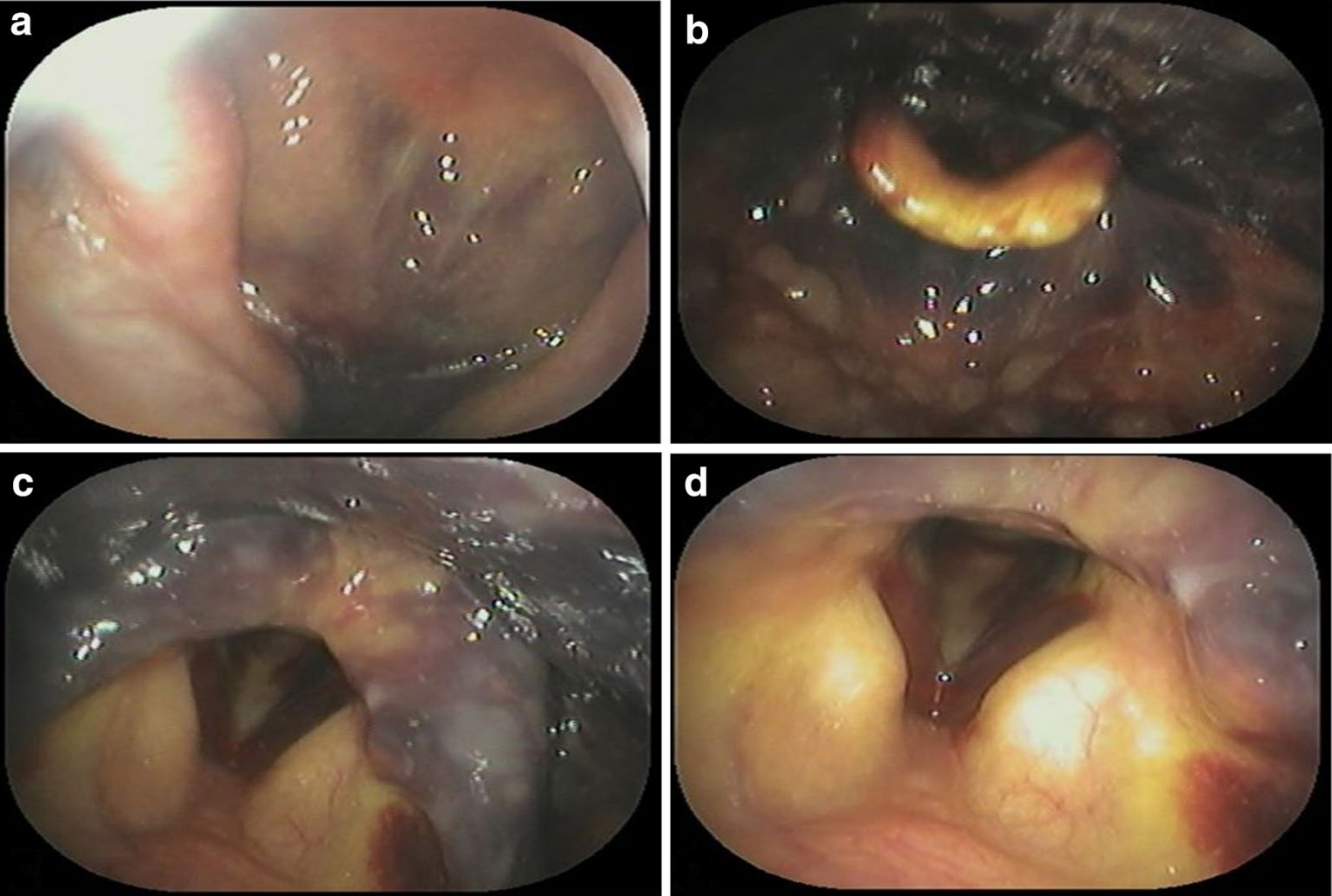
Case 1. Fiberoptic laryngoscopy. **a** Nasopharynx, **b** oropharynx, **c**, **d** larynx, submucous bleeding of the true vocal cords is evident

**Fig. 2:**
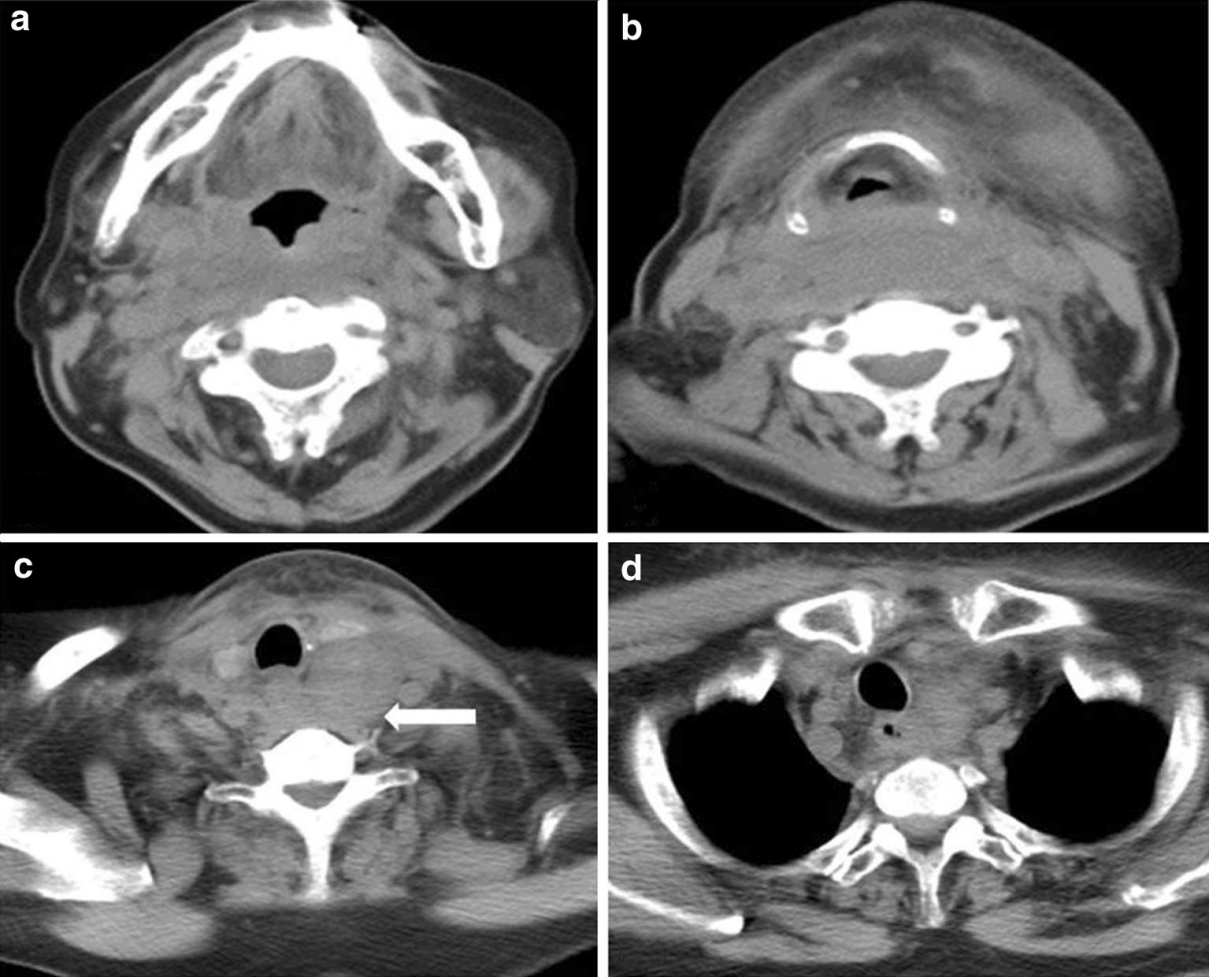
Case 1. Axial images of a CT of the neck and chest without contrast demonstrate diffuse soft tissue density posterior to the oropharynx, hypopharynx and larynx, displacing these structures anteriorly away from the spine (**a**, **b**). A more focal mass-like area of rounded soft tissue density is seen posterior to the left thyroid lobe, suggestive of a focal lesion (*arrow*) (**c**). Diffuse abnormal soft tissue density is also seen within the upper mediastinum (**d**)

**Fig. 3:**
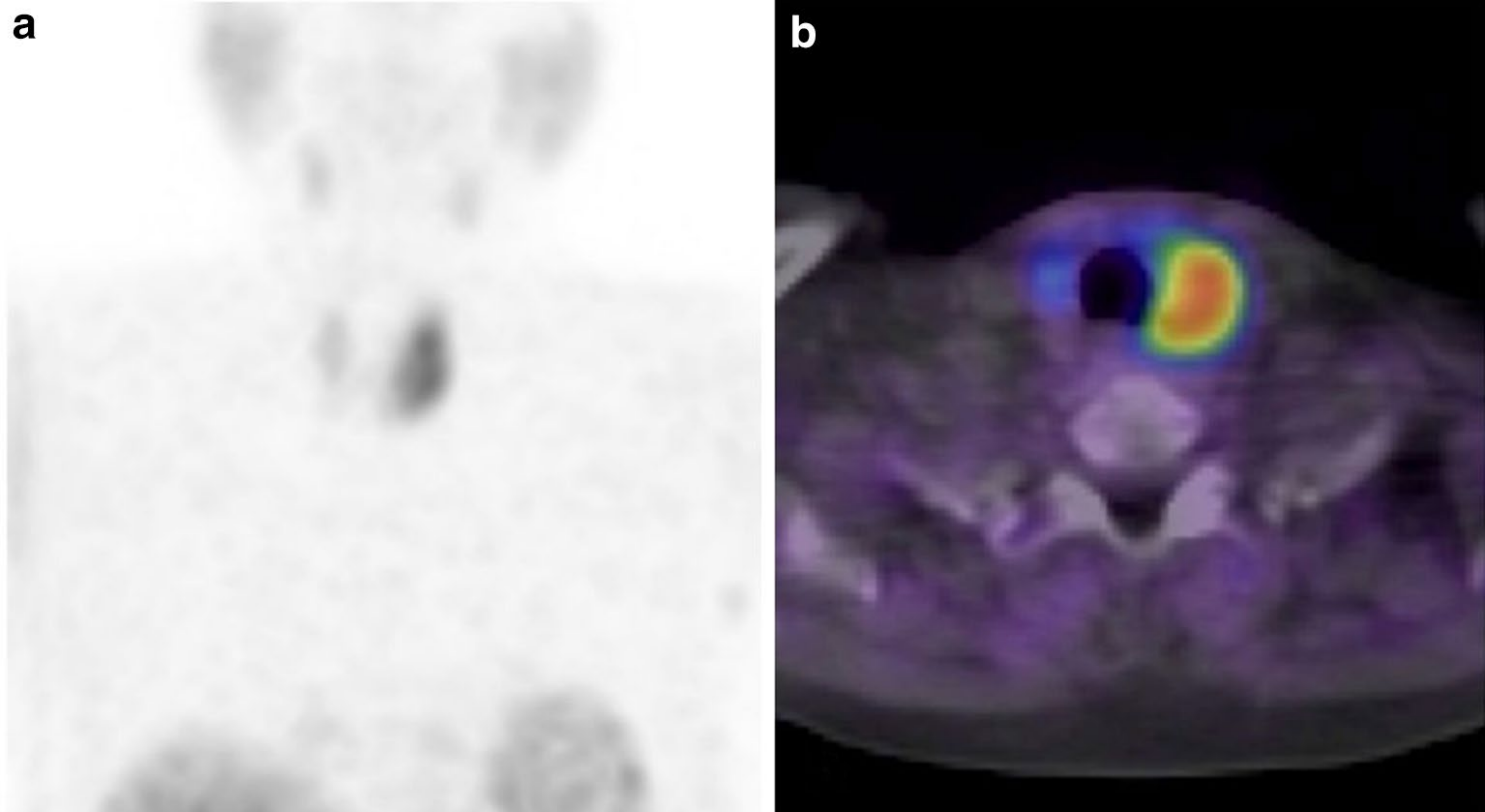
Case 1. **a** Delayed image of 99mTc-MIBI (99m-technetium-ethoxysobutylisonitrile) scintigraphy showing abnormal uptake on the left side of the parathyroid area, **b** SPECT-CT showing abnormal uptake in the same location

A 76-year-old Asian woman presented with pharyngeal discomfort and extensive ecchymosis over the neck and upper anterior chest wall. She had no history of trauma, but had been taking an aspirin for the treatment for internal jugular vein thrombosis. Endoscopic investigation revealed submucosal hemorrhage in the larynx and posterior wall of the nasopharynx, extending to the hypopharynx (Fig. 1). Her serum calcium level was 11.6 mg/dl (normal range 8.8–10.1 mg/dl) and measured intact PTH level was 453 pg/ml (normal range 10–60 pg/ml). Neck and chest CT scan confirmed the presence of a soft-density area extending from the retropharyngeal space to the mediastinum and a mass-like area (25 × 92 × 50 mm) behind the lower one-third of the left thyroid lobe (Fig. 2). 99 mTc-MIBI scintigraphy and SPECT-CT showed abnormal uptake in the left lower parathyroid gland (Fig. 3). A tentative diagnosis of hemorrhagic parathyroid adenoma resulting in retropharyngeal and mediastinal hematoma was made.

**Fig. 4:**
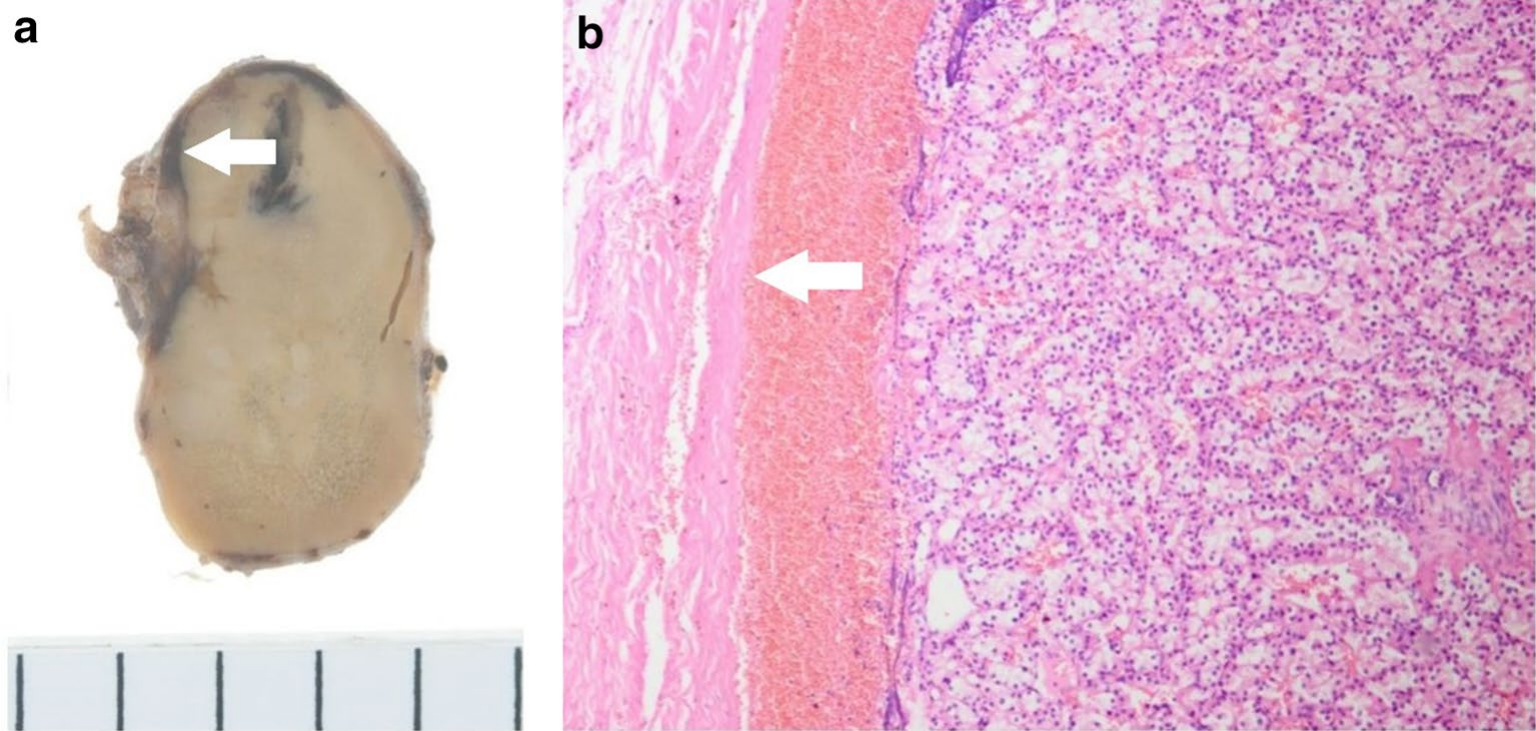
Case 1. **a** Macro specimen (cut surface) 35 × 25 × 15 mm slight hemorrhagic area is detected right under the capsule (*arrow*), **b** HE stain ×40. The tumor is surrounded by fibrous capsule (*arrow*)

The left lower parathyroid was removed 7 months after initial presentation. Through a collar skin incision a tumor was observed, situated caudal to the inferior pole of the left thyroid lobe, medial to the left common carotid artery, lateral to the esophagus, and overlying the left recurrent nerve. The tumor was elliptical, measuring 35 × 25 × 15 mm (Fig. 4), and adhered tightly to the surrounding organs. On the third postoperative day, the patient’s serum calcium level had become normal at 9.0 mg/dl and intact PTH level had decreased to 100 pg/ml. Two years after surgery, there has been no recurrence of hypercalcemia.

### Case 2

**Fig. 5:**
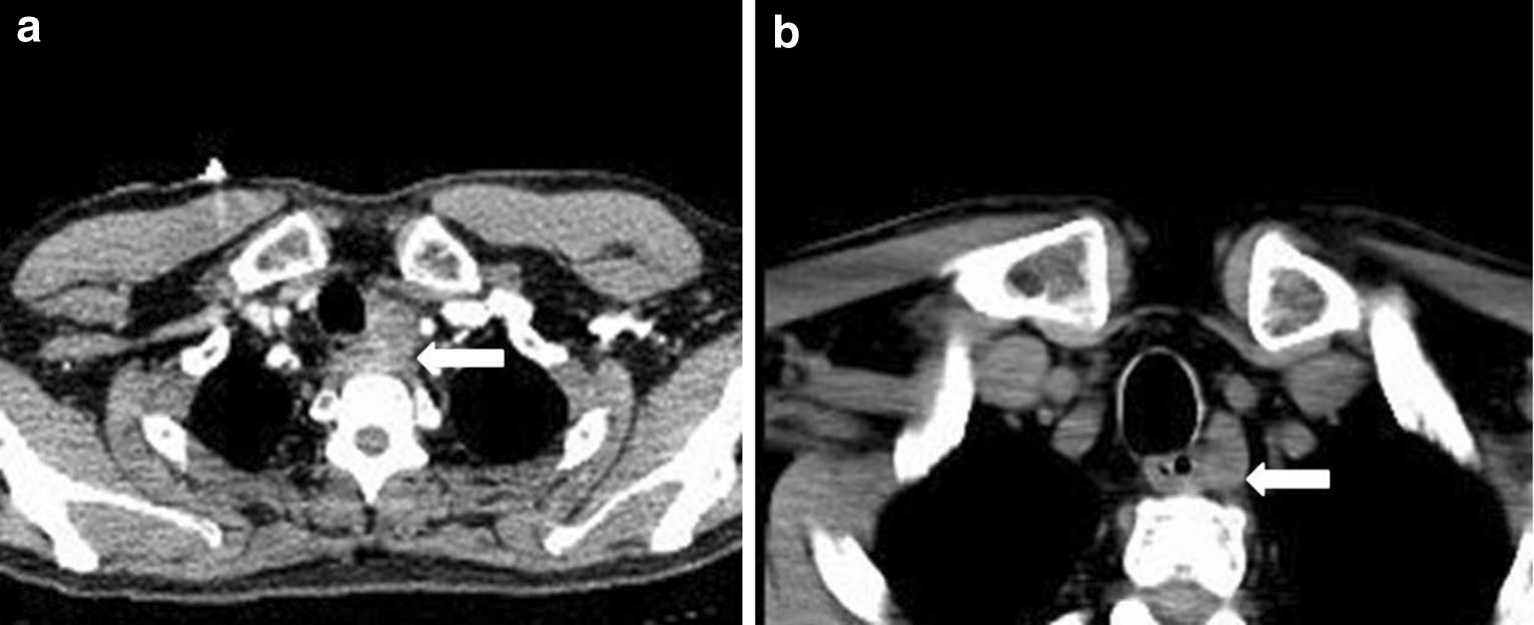
Case 2. **a** Axial image of a CT of the neck and chest with contrast demonstrate diffuse soft tissue density (*arrow*) in the posterior mediastinal space. **b** Axial image of a CT of the neck and chest without contrast examined 2 weeks after admission. The diffuse lesion has disappeared and a mass (*arrow*) posterior to the thyroid can be clearly seen

**Fig. 6:**
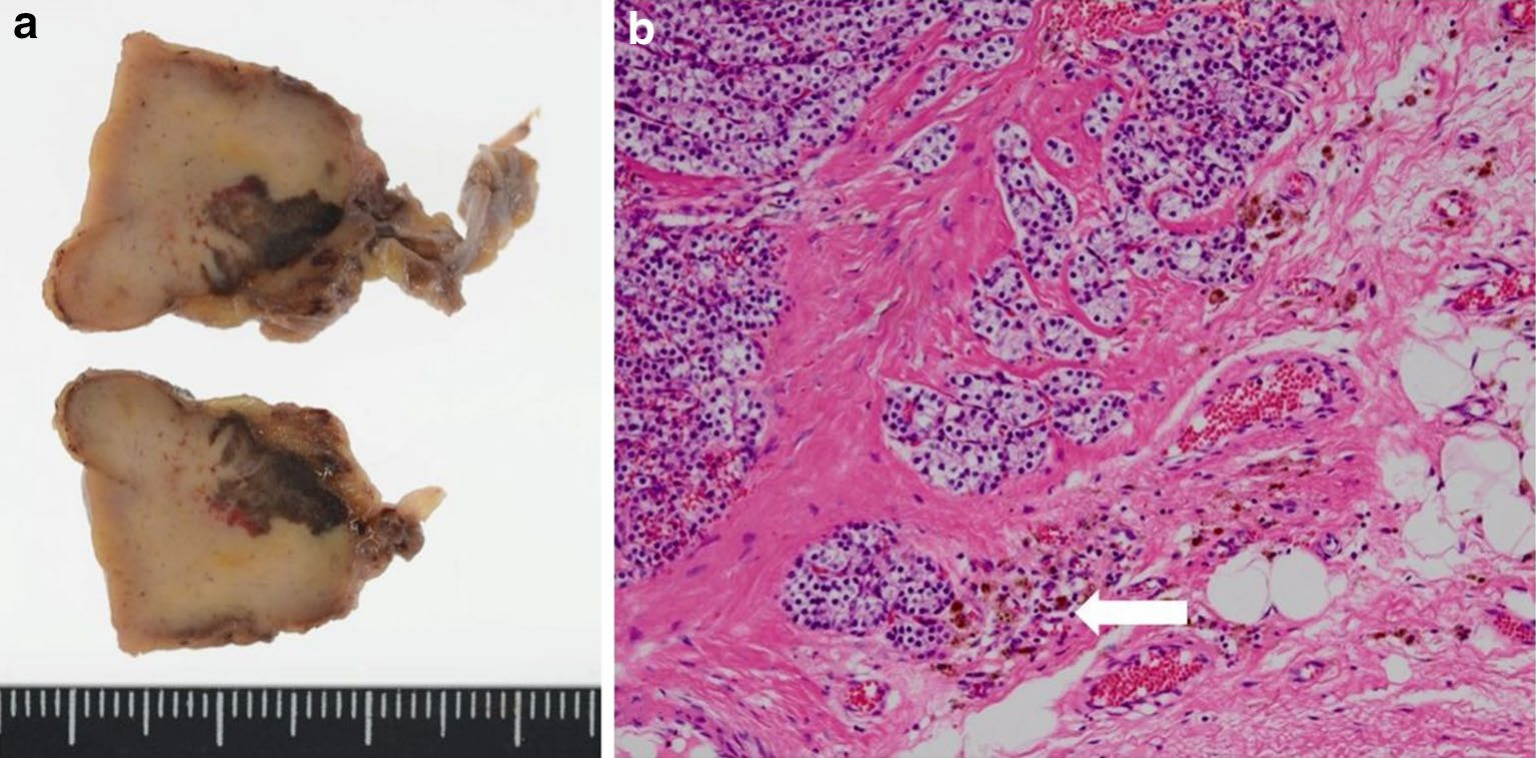
Case 2. **a** Macro specimen (cut surface) 28 × 19 × 17 mm hemorrhagic area is evident. **b** HE stain ×400. Phagocytosis of hemosiderin by macrophages is evident (*arrow*)

A 62-year-old Asian man presented with anterior chest wall ecchymosis in the absence of any prior injury. CT scan demonstrated an area of soft density next to the esophagus (Fig. 5a), and a hematoma due to bleeding from an upper mediastinal lesion or a ruptured blood vessel was suspected. The patient was closely followed up in the ICU, and underwent daily CT and blood examinations. The ecchymosis gradually improved, and the mediastinal mass also became smaller within a week. Two weeks after admission, CT scan demonstrated the presence of a mass below the left thyroid lobe which appeared to be contiguous to the previously observed hematoma (Fig. 5b). The patient’s serum calcium level was 11.7 mg/dl (normal range 8.8–10.1 mg/dl) and the intact PTH level was 201 pg/ml (normal range 10–60 pg/ml). The left lower parathyroid was removed 7 months after initial presentation. The tumor, measuring 28 × 19 × 17 mm, was located caudal to the inferior pole of the left thyroid lobe, and adhered to surrounding tissues including the esophageal muscle and recurrent nerve. Phagocytosis of hemosiderin by macrophages was evident, suggesting hemorrhage from a parathyroid adenoma (Fig. 6). On the second postoperative day, the patient’s serum calcium level had improved to 9.5 mg/dl and the intact PTH level to 13 pg/ml.

## Discussion

Neck hematoma is relatively rare and mostly a secondary consequence of infection, cervical spine trauma, great vessel trauma, iatrogenic injury associated with catheterization and angiography, or ingestion of a foreign body. Spontaneous neck hematoma also can occur as a result of thyroid or parathyroid extraglandular bleeding. The causative thyroid lesions include thyroid cyst, nodular goiter and subacute thyroiditis, whereas causative parathyroid lesions include adenoma, hyperplasia, cysts and cancer. Anticoagulation or hemorrhagic diathesis may be a predisposing factor in such cases [7].

Hemorrhage into a thyroid cyst is not uncommon, being usually palpable and quite tender, but bleeding into surrounding areas is rare because the thyroid capsule is relatively thick. Subacute thyroiditis following a viral illness can cause many systemic symptoms and a characteristically high sedimentation rate without any change in the level of serum calcium. Parathyroid bleeding is most often seen in patients with adenoma [8]. As neck hematoma can easily spread to the mediastinum and pleural space resulting in a life threatening condition, practitioners should be aware of other potentially critical sources of mediastinal hematoma, such as aortic dissection and mediastinal blood vessel rupture.

Extraglandular hemorrhage from a parathyroid adenoma is a very rare form of neck hematoma. In a review of the world literature and a series of cases from their center, Chaffanjon et al. identified only four hemorrhagic parathyroid adenomas out of a total of 692 cases [9]. Extracapsular parathyroid hemorrhage may occur when the parathyroid glands are enlarged due to tumors such as adenomas, primary or secondary hyperplasia, cysts, or cancers [10]. Expansion outside the capsule may be due to the fact that parathyroid glands containing tumors have relatively thin and weak capsules. As mentioned above, anticoagulation or hemorrhagic diathesis may aggravate the development of neck hematoma. In Case 1, the patient had been treated with an anticoagulant drug for internal jugular vein thrombosis.

The symptoms and signs of hematoma from a parathyroid tumor depend on the anatomical position of the tumor, the amount of bleeding and the increase or decrease in secretion of PTH accompanying the infarction and/or bleeding in the gland [10].

Cervical hematoma caused by a parathyroid tumor is characterized by painful swallowing, dysphagia, dyspnea, hoarseness, swelling of the anterior neck, or ecchymosis of the neck or chest [2, 10–13]. Severe compression of the pharynx or larynx can lead to narrowing of the airway, and may require emergency tracheostomy. Compression of the recurrent laryngeal nerve by a cervical hematoma can cause vocal cord paralysis. In particular, hoarseness, dysphonia and acute respiratory failure can be warning symptoms [14, 15].

Mediastinal hematoma or hemothorax due to intrathoracic parathyroid tumor bleeding is characterized by chest pain, cough, shortness of breath, or respiratory distress. Moreover, if the hemothorax is bilateral, it can induce acute severe respiratory failure [16]. The patient may also become hypotensive because of compression of the large vessels. Finally, mediastinal hematoma from a parathyroid tumor may mimic rupture of an aortic aneurysm [17, 18], as seen in Case 2.

Simcic and McDermott proposed three diagnostic criteria for hemorrhaging parathyroid tumors: acute neck swelling, hypercalcemia, and ecchymosis of the neck or chest [19]. After the introduction of these criteria, many cases lacking part of this triad were reported. As aortic dissection and blood vessel rupture can also cause sudden swelling and ecchymosis, hypercalcemia and the presence of a nodule behind the thyroid are the most specific symptoms of parathyroid bleeding. Sometimes, however, parathyroid adenoma can be non-functional for three reasons. First, there may be tissue necrosis secondary to cystic degeneration. Second, PTH may be secreted into the lumen of the cyst instead of the bloodstream. Third, the pressure caused by the hematoma may interfere with blood flow around the adenoma [20].

When encountering cervical and/or mediastinal hematoma, blood testing, which can detect hypercalcemia and a high level of intact PTH, is one of the key examinations for investigating the possibility of parathyroid adenoma bleeding. Imaging techniques such as ultrasonography, CT and MRI can identify nodular structures and differentiate solid tissue from cysts, but it may sometimes be difficult to distinguish between a thyroid nodule, a lymph node, and a parathyroid adenoma. However, scintigraphy (Tc-sestamibi imaging) can be used to distinguish between thyroid and parathyroid tissue and identify overactive parathyroids [4, 21]. The treatment of bleeding from a parathyroid adenoma requires surgical exploration and excision of the tumor after appropriate localization. The optimal timing of surgery remains controversial, however. For most previously reported cases, parathyroidectomy was performed within several weeks after the occurrence of hemorrhage. In some cases, however, the tumors were excised incompletely, and the recurrent laryngeal nerve was injured. Chaffanjon et al. proposed that if there are no severe compressive or hemodynamic symptoms, surgery should be performed more than 3 months after the occurrence of hemorrhage because the dissection then becomes as simple as for any other form of planned surgery [9]. Nevertheless, patient who presents with hemorrhage from a parathyroid tumor should not be observed for too long without excision of the tumor because there is a possibility that the hemorrhage will recur.

## Conclusions

Although hemorrhage from a parathyroid adenoma is very rare, it can be one condition responsible for sudden swelling and ecchymosis of the neck and anterior chest in the absence of any precipitating trauma. For an accurate diagnosis, examination of blood test results for calcium and PTH, CT and/or MRI is essential.

## Consent

Written informed consent was obtained from the patients for publication of this case report and any accompanying images.

## Abbreviation

PTH: parathyroid hormone
